# pH-Controlled fluorescence switching in water-dispersed polymer brushes grafted to modified boron nitride nanotubes for cellular imaging

**DOI:** 10.3762/bjnano.10.233

**Published:** 2019-12-10

**Authors:** Saban Kalay, Yurij Stetsyshyn, Volodymyr Donchak, Khrystyna Harhay, Ostap Lishchynskyi, Halyna Ohar, Yuriy Panchenko, Stanislav Voronov, Mustafa Çulha

**Affiliations:** 1 Department of Genetics and Bioengineering, Yeditepe University, Atasehir, 34755 Istanbul, Turkey; 2 Lviv Polytechnic National University, 12 S. Bandery, 79013 Lviv, Ukraine

**Keywords:** boron nitride nanotubes, cellular imaging, fluorescence, pH switching, polymer brushes, surface modification

## Abstract

pH-Switchable, fluorescent, hybrid, water-dispersible nanomaterials based on boron nitride nanotubes (BNNTs) and grafted copolymer brushes (poly(acrylic acid-*co*-fluorescein acrylate) – P(AA-*co*-FA)) were successfully fabricated in a two-step process. The functionalization of BNNTs was confirmed by spectroscopic, gravimetric and imaging techniques. In contrast to “pure” BNNTs, P(AA-*co*-FA)-functionalized BNNTs demonstrate intense green fluorescence emission at 520 nm. Under neutral or alkaline pH values, P(AA-*co*-FA)-functionalized BNNTs are highly emissive in contrast to acidic pH conditions where the fluorescent intensity is absent or low. No increase in the absorption was observed when the suspension pH was increased from 7 to 10. The functionalized BNNTs are easily taken up by human normal prostate epithelium (PNT1A) and human prostate cancer cell lines (DU145) and are suitable for further evaluation in cellular imaging applications.

## Introduction

In recent years, considerable effort has been devoted to the development of hybrid nanomaterials [1–5] to generate novel structures with tunable properties through external stimuli such as pH, temperature, light, and magnetic field [6–10]. Among other nanomaterials, significant research effort has been dedicated to the use of nanotubes [1–4,6–7,11–16]. For the last two decades, carbon nanotubes [17], such as metal nanotubes [18], oxide nanotubes [19], boron nitride nanotubes (BNNTs) [12] and nanotubular clays [6–7] were intensively studied. The reason behind the widespread interest in nanotubes is due to their excellent application potential in several fields [1–4,6–7,11–16]. BNNTs were first synthesized by Chopra et al. [20] in 1995 and they are considered as the structural analog to CNTs. BNNTs are of particular interest due to their remarkable mechanical properties (e.g., Young’s modulus of 1.22 TPa) and low toxicity [21–24]. They are superhydrophobic in their pristine state and cannot be dispersed in aqueous media or in most organic solvents [11,22–23]. To improve the dispersibility of BNNTs in aqueous media, numerous surface functionalization methods have been used [11,25–27]. The functionalization strategies can be classified into three groups: (1) noncovalent, (2) covalent grafting to the surface, and (3) formation of amino and/or hydroxyl groups at the ends and defect sites [11,25–27]. Up to now, the most prospective procedure for BNNT functionalization is covalent grafting of polymer brushes via polymerization initiated from their surface [12,28]. In particular, BNNTs were covalently modified with hydrophobic polystyrene or poly(glycidyl methacrylate) polymer brushes [28]. The modified nanotubes demonstrated high dispersibility in a large number of organic solvents. In our previous work [12], BNNTs were functionalized with thermo-responsive poly-*N*-isopropylacrylamide (PNIPAM) and became dispersible in water. The hydrodynamic radius of PNIPAM-modified BNNTs was decreased two-fold at temperatures above 32 °C. Along with other nanomaterials, the various nanotubes were tested as fluorescent probes for a number of bio-responsive applications, ranging from drug delivery to genomics [29–31]. Similar to other nanotubes, the pristine BNNTs were not fluorescent, and a fluorophore (e.g., organic molecule or quantum dot) is added via surface modification to make them fluorescent [29–34].

In [35], the fluorescent CdSe quantum dots were attached to BNNT surfaces, and in [36] the halloysite nanotubes were modified with carbon dots and used for cellular imaging. Another approach is surface modification with grafted polymers bearing organic fluorophores. One of the most commonly used labels is fluorescein [37–42]. In biomedical applications, fluorescein has several advantages over other dyes such as nontoxicity, high water solubility, and pH responsivity. Fluorescein demonstrates a high fluorescence efficiency at basic pH values but becomes nonfluorescent under acidic conditions [42].

In our previous work [12], we have reported a two-step method for preparation of a responsive surface layer on oligoperoxide-functionalized BNNTs, where amino groups on BNNTs form at the ends and defects. Additional amine groups can be easily introduced with ammonia plasma irradiation [43], by mechanical milling [44] or with the iminoborane treatment [45]. The first step in covalent bonding of oligoperoxide to BNNT surfaces is to bind pyromellitic chloroanhydride moieties through available amino groups. The second step involves the grafting polymerization “from the surface” of oligoperoxide-functionalized BNNTs. With this procedure, we have created a hybrid nanomaterial for cellular imaging based on BNNTs and grafted brushes of pH-responsive fluorescent copolymer poly(acrylic acid-*co*-fluorescein acrylate) – P(AA-*co*-FA). The functionalization of BNNTs was studied with thermogravimetric analysis (TGA), Fourier transform infrared spectroscopy (FTIR), dynamic light scattering (DLS), ultraviolet–visible spectrophotometry (UV–vis), laser scanning confocal microscopy (LSCM) and scanning electron microscopy (SEM). The DLS results demonstrated that P(AA-*co*-FA)-functionalized BNNTs exhibited good dispersibility in water. pH-Controlled switching of fluorescence was demonstrated using LSCM and UV–vis. In addition, the potential of BNNTs as novel pH-switchable fluorescent water-dispersible materials for cellular imaging has been investigated. The developed hybrid structure can potentially be used not only for cellular imaging but also as “smart” surfaces, nanotransducers and nanocarriers.

## Experimental

### Materials

Pyridine and other organic solvents were purified as reported by Riddick et al. [46]. Poly(ethylene glycol) (PEG-9) was supplied by Merck Chemical Co.; acrylic acid and fluorescein acrylate were supplied by Sigma-Aldrich. Colemanite (Ca_2_B_6_O_11_·5H_2_O) was obtained from ETI Mine Works General Management (Turkey). Iron(III) oxide, hydrochloric acid, and nitric acid were purchased from Sigma-Aldrich. Highly pure NH_3_ gas (99.98%) was provided by Schick GmbH & Co. KG. All solutions were prepared with distilled water.

### Synthesis

#### Synthesis of *tert*-butyl hydroperoxide

*tert*-Butyl hydroperoxide was obtained as described in [47] and purified by vacuum rectification. The fraction, boiled in the temperature range of 45–47 °C (at 1.6 kPa), was collected. Its refractive index was determined to be *n*_d_^20^ = 1.40020 ± 0.00002, which is in agreement with values reported elsewhere (*n*_d_^20^ = 1.4010) [47].

#### Synthesis of pyromellitic acid chloride

As described in [12], in a 500 mL round-bottomed flask equipped with a thermometer and a reflux condenser connected with water scrubber, 43.6 g (0.2 mol) of pyromellitic dianhydride and 91.6 g (0.44 mol) of PCl_5_ were mixed and boiled in the oil bath until the mixture became homogeneous. The mixture was stirred at 130–135 °C for 15–16 h. Then, the reflux condenser was replaced by a Liebig condenser, and approximately 60–63 g of POCl_3_ was distilled out during 8 h. At the end of the distillation, the temperature of the mixture was increased up to 180–185 °C for 1 h. The crude product was recrystallized from gasoline, yielding 51.2 g (78.1%) of a colorless crystalline product with the melting point of 67 °C (literature value 68 °C) [48] and the acid number AN = 1373 mg KOH/g (theoretical value is 1368 mg KOH/g).

#### Synthesis of oligoperoxide with residual acid chloride groups

As described in [12], 4.6 g (0.014 mol) of pyromellitic acid chloride was dissolved in 15 mL of anhydrous dichloroethane, placed in a three-necked flask equipped with a stirrer and 1.26 g (0.014 mol) of *tert*-butyl hydroperoxide was added. The mixture was cooled down to 5 °C and then 1.1 g (0.014 mol) of pyridine, dissolved in 10 mL of anhydrous dichloroethane, was added dropwise at 5 °C. The suspension was stirred for 1 h. Subsequently, 5.6 g (0.014 mol) of PEG-9 was added, and again the solution of 2.2 g (0.028 mol) pyridine in 10 mL of anhydrous dichloroethane was admixed dropwise. The mixture was then stirred for another 3 h, and the temperature was increased gradually to ambient temperature. The pyridinium chloride precipitate was filtered out. The solvent was distilled out and the pellet was dried in vacuum (100–200 Pa) at 40 °C for 3 h, yielding 8.2 g (81%) of oligoperoxide. Its characteristics are summarized as follows: content of active oxygen – 1.9% (calc. 2.2%); content of active chlorine – 5.4% (calc. 4.9%); AN = 163.1 mg KOH/g (calc. 155.3 mg KOH/g); characteristic infrared spectra bands – ν (С=О) in Ar-C(O)Cl, ν (С=О) in ester group at 1760 and 1752 cm^−1^; doublet at 1390, 1365 cm^−1^, referred to δ(C(CH_3_)_3_) and a band of *tert*-butoxy group at 848 cm^−1^.

#### BNNT synthesis and purification

The BNNTs were synthesized by chemical vapor deposition from colemanite (2 g) and Fe_2_O_3_ (0.166 g) [25]. Briefly, the precursor mixture was transferred to an alumina boat and preheated at 180 °C for 15 min and then the alumina boat was placed into the center of the tubular furnace (Protherm, Furnaces PTF 14/50/450). The BNNT synthesis was performed under saturated NH_3_ atmosphere. The furnace temperature was set to a heating rate of 8 °C/min up to 1280 °C and then held at this temperature for 3 h. The furnace was cooled down to ambient temperature. Thereafter, the BNNTs were collected from the top of the alumina boat and stored in a dry environment.

For the purification, the obtained BNNTs were added to 50 mL 4 M HCl, stirred for 4 h at 90 °C and then collected by centrifugation (14000 rpm, 30 min). The obtained product was stirred in 30 mL of 1 M HNO_3_ for 6 h at 30 °C and was then collected by centrifugation at 14000 rpm for 30 min. After the centrifugation, the BNNTs were washed with distilled water until all acid was removed and were then dried at 60 °C overnight.

### Modification of BNNTs

#### Modification of BNNTs with oligoperoxide

**Scheme 1:**
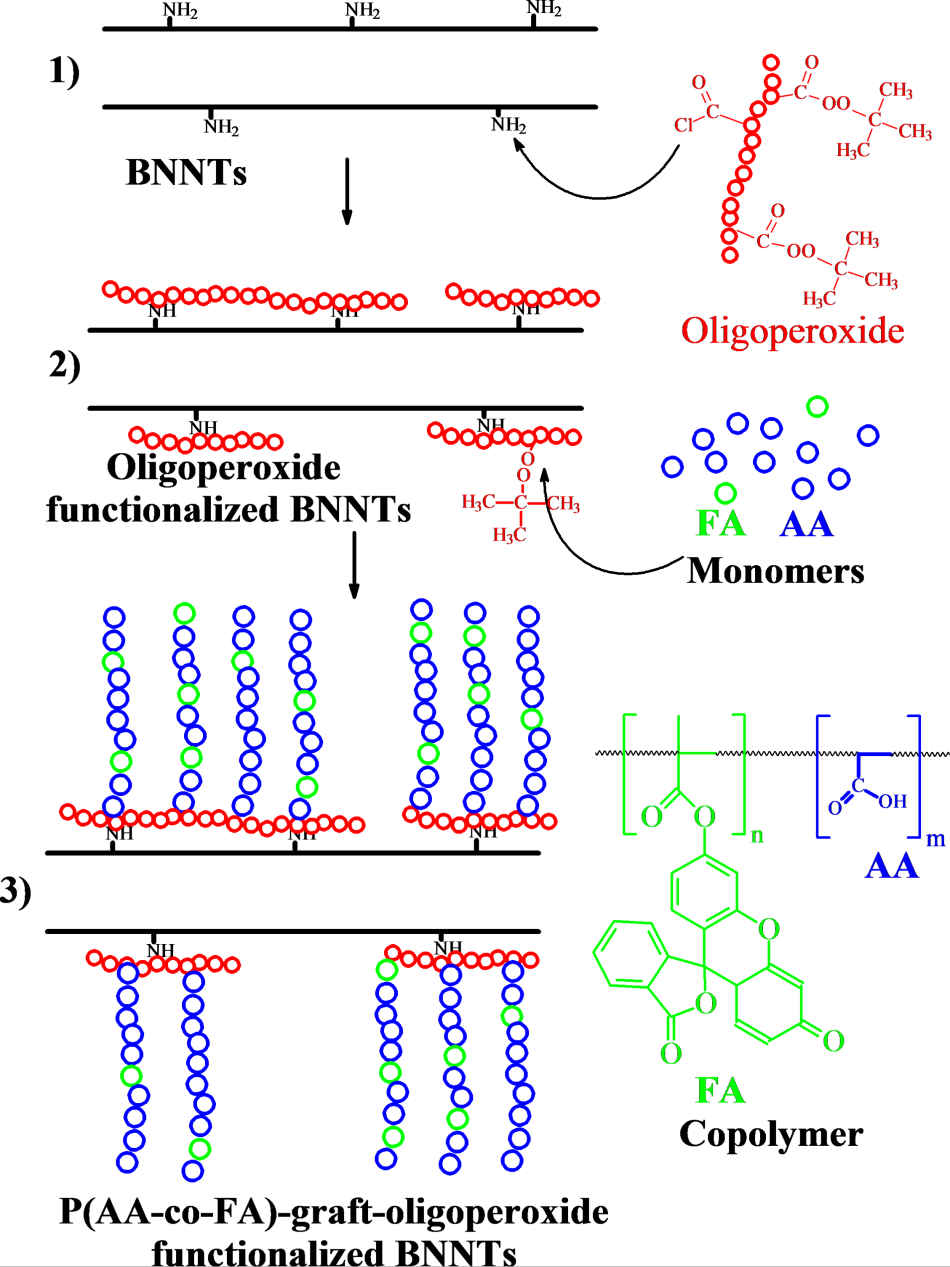
BNNT (1) functionalization with oligoperoxide (2), and subsequent grafting copolymerization of acrylic acid (AA) and fluorescein acrylate (FA) (3), initiated by peroxide groups of oligoperoxide and resulting in P(AA-*co*-FA)-functionalized BNNTs.

The modification procedure of BNNTs is presented below in Scheme 1. As described in [12], 50 mg of powdered BNNTs (1) were mixed with 50 mL of oligoperoxide solution in anhydrous dioxane (1 mg/mL) and sonicated for 20 min after the solution was stirred vigorously for 24 h. The excess of oligoperoxide was removed by repeated dispersion with sonication into dioxane and toluene and collection of the nanotubes by centrifugation. The purified oligoperoxide-functionalized BNNTs were dried and used for grafting polymerization (2).

#### Fabrication of P(AA-*co*-FA)-grafted brushes

BNNTs with grafted oligoperoxide [(2) below in Scheme 1] were placed in a container with 0.1 М of AA and 0.01 M FA aqueous solution and sonicated for 20 min. Then, the mixture was heated under argon atmosphere to 90 °C for 48 h, resulting in the P(AA-*co*-FA)-graft-oligoperoxide functionalized BNNTs (3). These were purified by repeated dispersed in ethanol and water with sonication and collection of the nanotubes by centrifugation. The purified P(AA-*co*-FA)-graft-oligoperoxide functionalized BNNTs were dried and stored as a powder.

### Characterization of BNNTs

#### Thermogravimetric analysis (TGA)

TGA analysis was performed using a Perkin Elmer Pyris 1 TGA instrument with Thermal Analysis Gas Station and Pyris Version 11.1.1.0497 software. The samples were analyzed under N_2_ gas flow, which was heated from 30.00 to 600.00 °C at 10 °C/min.

#### Fourier transform infrared spectroscopy (FTIR)

FTIR spectra were recorded on a NICOLET iS50 FTIR spectrometer (Thermo Scientific, USA), equipped with a iS50 ATR multirange, diamond sampling station. The sample powders were either placed on diamond crystal or mixed with KBr to form a pellet. The spectra in the range of 7800–350 cm^−1^ with a 5 cm^−1^ step were collected in reflection or in transmission mode.

#### Laser scanning confocal microscopy (LSCM)

P(AA-*co*-FA)-functionalized BNNTs were imaged with a Zeiss Axio Imager M2 laser scanning confocal microscope (Carl Zeiss Microimaging GmbH, Zeiss, Jena, Germany), equipped with the following optical elements: Zeiss 5×/0.16 EC Plan-NEOFLUAR, Zeiss 10×/0.13 EC Plan-NEOFLUAR, Zeiss 20×/0.8 Plan-Apochromat, Zeiss 40×/0.95 Kor, Zeiss 63×/1.4 oil DIC Plan-Apochromat and Zeiss 100×/1.4 oil DIC Plan-Apochromat. In our study the excitation and emission wavelengths were selected at 490 nm and 520 nm, respectively, and the fluorescently labeled nanomaterial was viewed.

#### Ultraviolet–visible spectrometry (UV–vis)

The optical absorption spectra of the BNNT suspension were registered on a double-beam UV–vis spectrophotometer (Lambda 35, PerkinElmer, Germany) by scanning in the range of 300–900 nm at room temperature. Prior to the measurement, the baseline was recorded for a cuvette with pure solvent. The nanotube concentration was 1 mg/mL, and the pH was adjusted by adding HCl or NaOH.

#### Fluorescence spectrophotometry

Fluorescence emission spectra were recorded on a Varian Eclipse spectrophotometer using a 1 cm path length cuvette at room temperature. The excitation and emission were scanned simultaneously at wavelengths from 200 to 600 nm and from 220 to 600 nm, respectively, at 5 nm intervals, with a 5 nm slit width at a 9600 nm/min scan rate. The excitation and emission wavelength and the maximum fluorescence intensity of each peak were recorded. The concentration of BNNTs was 1 mg/mL. The pH of the suspension was adjusted by adding HCl or NaOH.

#### Dynamic light scattering (DLS)

**Figure 1:**
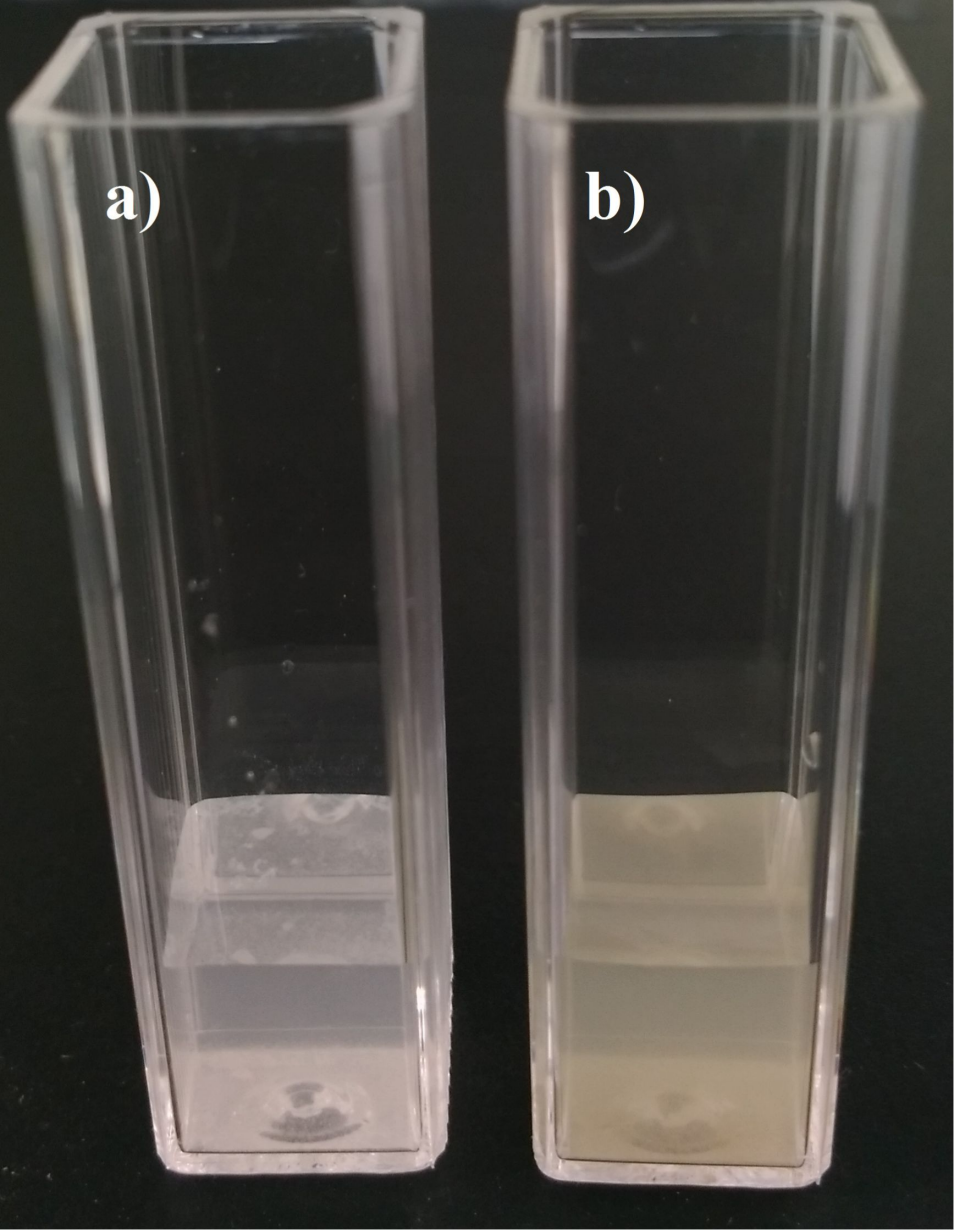
Suspensions of native BNNTs (a) and P(AA-*co*-FA)-functionalized BNNTs (b) obtained at a concentration of 1 mg/mL in distilled water after 2 min of sonic bath treatment.

The size distribution and zeta potential measurements of the nanotubes were performed with a Zetasizer Nano ZS device (Malvern, USA) at 25 °C. The Nano ZS contains a 4 mW He–Ne laser operating at a wavelength of 633 nm and an avalanche photodiode detector. The scattered light was detected at an angle of 173°. The refractive index and absorption of the nanomaterial were assumed to be 2.0 and 1.500, respectively. All size and zeta potential measurements were carried out in triplicate. All data were analyzed by using Malvern Instrument DST 5.00 software. 1 mg of sample was suspended in 1 mL of distilled water and sonicated for 2–10 min. After the sonication, low speed (1000*g*, 5 min, 25 °C) centrifugation was carried out and the supernatant (see below Figure 1) was used for analysis.

#### Scanning electron microscopy (SEM)

The BNNTs were placed on a carbon disc and coated with a 5 nm thick gold layer using a Baltec SDC 005 sputter coater. SEM images were obtained with a Carl Zeiss Evo-40 instrument under high vacuum and accelerating voltage of 10 kV.

#### Cell culture experiments

Normal prostate epithelium (PNT1A) and human prostate cancer (DU145) cell lines were grown in Dulbecco’s Modified Eagle’s Medium, supplemented with 10% fetal bovine serum and 1% penicillin/streptomycin/ampicillin and incubated in a water-jacketed incubator in a 5% CO_2_, 95% air atmosphere at 37 °C. The cells were expanded in T-75 flasks to 80% confluence and then detached with trypsin and collected. A 1 mg/mL sample of P(AA-*co*-FA)-functionalized BNNTs dispersed in ultrapure water was used in cellular uptake studies. The cells were imaged with a Zeiss Axio Imager.M2 instrument at an excitation wavelength of 490 nm and emission of 520 nm.

## Results and Discussion

### Fabrication and properties of fluorescent boron nitride nanotubes

The pH-responsive fluorescent coatings were formed on the BNNT surface via a simple two-step process as outlined in Scheme 1 and described in detail in the Experimental section. The first step: a multifunctional radical initiator (oligoperoxide) was grafted to the BNNT surface by means of interaction of amino groups (at the defects on the BNNT surface) with chloroanhydride fragments in oligoperoxide molecules [12]. The second step involved grafting copolymerization of acrylic acid and fluorescein acrylate initiating “from the surface” of oligoperoxide-functionalized BNNTs to fabricate grafted polymer brushes.

The functionalized BNNTs were characterized using spectroscopic, gravimetric and imaging techniques including FTIR, UV–vis, DLS, TGA, LSCM and SEM. The fabrication of the oligoperoxide-functionalized BNNTs was described in detail in our previous publication [12] and here we focus on the synthesis and properties of the P(AA-*co*-FA)-functionalized BNNTs. The functionalized BNNTs after the copolymerization of acrylic acid and fluorescein acrylate has demonstrated an excellent dispersibility in water (Figure 1b) forming a suspension with a light-yellow color without the formation of any visible aggregates. In contrast, native BNNTs are not dispersed at all and white aggregates are visible on the top of the suspension and at the sides of the cuvette (Figure 1a).

To confirm the chemical modification and to isolate oligoperoxide and P(AA-*co*-FA)-graft-functionalized BNNTs from physically adhered polymers, several cycles of centrifugation/decanting/redispersion in different solvents and water were repeated (see Experimental part).

**Figure 2:**
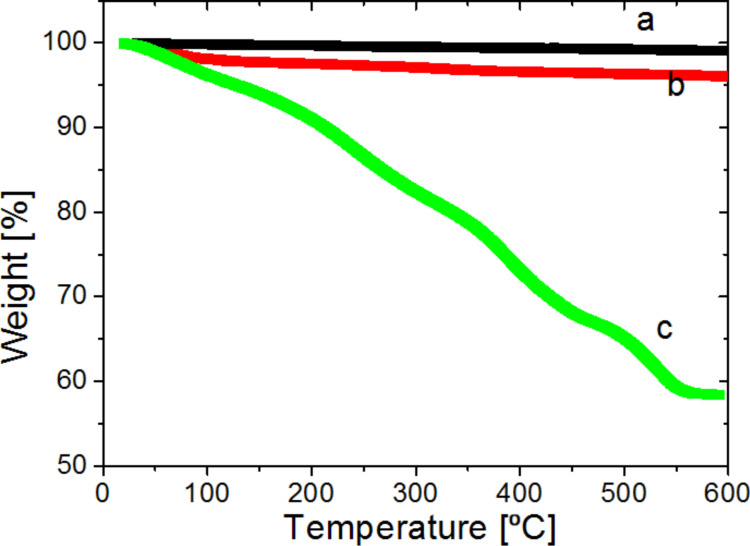
TGA curves of pristine BNNTs (a), oligoperoxide-functionalized BNNTs (b) and P(AA-*co*-FA)-functionalized BNNTs (с).

Figure 2 shows the TGA curves of native BNNTs (a), and oligoperoxide-functionalized BNNTs (b) and P(AA-*co*-FA)-functionalized BNNTs. The weight loss of oligoperoxide-functionalized BNNTs (Figure 2, curves b) is relatively low and is only a few percent. In contrast, P(AA-*co*-FA)-functionalized BNNTs demonstrate substantial mass loss that is nearly 60%. P(AA-*co*-FA)-functionalized BNNTs exhibit a middle thermal stability around 180 °C and drastic degradation from 180 to 550 °C. A low concentration of the oligoperoxide macromolecules covalently grafted to BNNTs suggests that not all oligoperoxide macromolecules covalently bound and some remain electrostatically attached to the nanotube surface and are washed off during the functionalization procedure. It also suggests that P(AA-*co*-FA)-grafted brushes grow vertically from the surface of the BNNTs functionalized by oligoperoxide.

**Figure 3:**
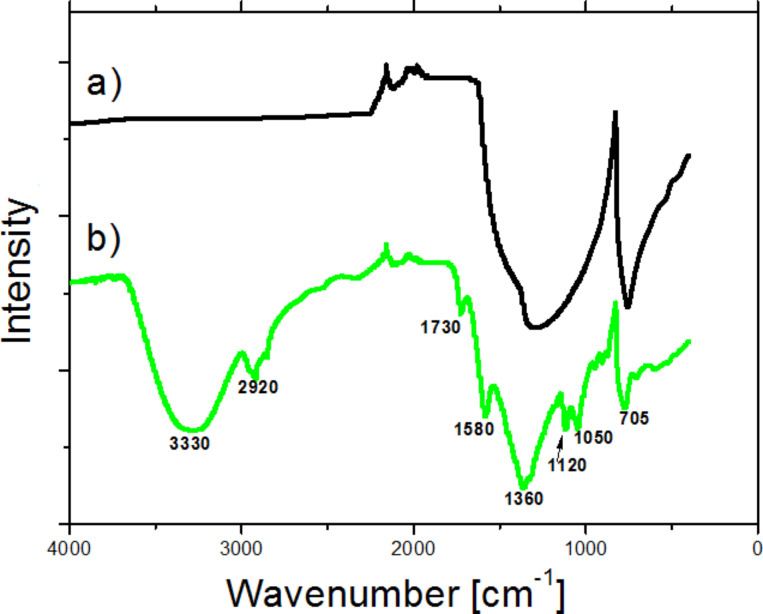
FTIR spectrum of pure BNNT (a) and P(AA-*co*-FA)-functionalized BNNTs (b).

The recorded FTIR spectra of pristine and P(AA-*co*-FA)-functionalized BNNTs are presented in Figure 3. The spectrum of pristine BNNTs is comprised of two broad asymmetric absorption bands, the B–N–B longitudinal in-plane bond vibration at 1327 cm^−1^, and the out-of-plane radial buckling of B and N atoms at 758 cm^−1^ (Figure 3a, black line), which is in full agreement with an earlier report. The FTIR spectra of oligoperoxide-modified BNNTs was described in detail in our previous paper [12] and is not discussed here. After the fabrication of the P(AA-*co*-FA)-functionalized BNNTs, a number of new bands, originating from grafted P(AA-*co*-FA) fragments, were revealed. New absorption bands at 1327 cm^−1^ and 758 cm^−1^ were observed. A broad absorption band at 3330 cm^−1^ is assigned to the –OH group of fluorescein acrylate and acrylic acid. Another band at 2920 cm^−1^ is attributed to stretching vibrations of C–H bonds. The absorption band at 1730 cm^−1^ is formed by carbonyl fragment in carboxyl and ester groups of the fluorescein and acrylic acid. A characteristic peak of benzene rings of fluorescein acrylate appears at around 1580 cm^−1^. In addition, the С–О–С bonds in esteric fragments are revealed by the stretching vibrations at 1120 and 1050 cm^−1^. All of this spectral information confirms the successful fabrication of grafted P(AA-*co*-FA) brushes on oligoperoxide-functionalized BNNTs.

**Figure 4:**
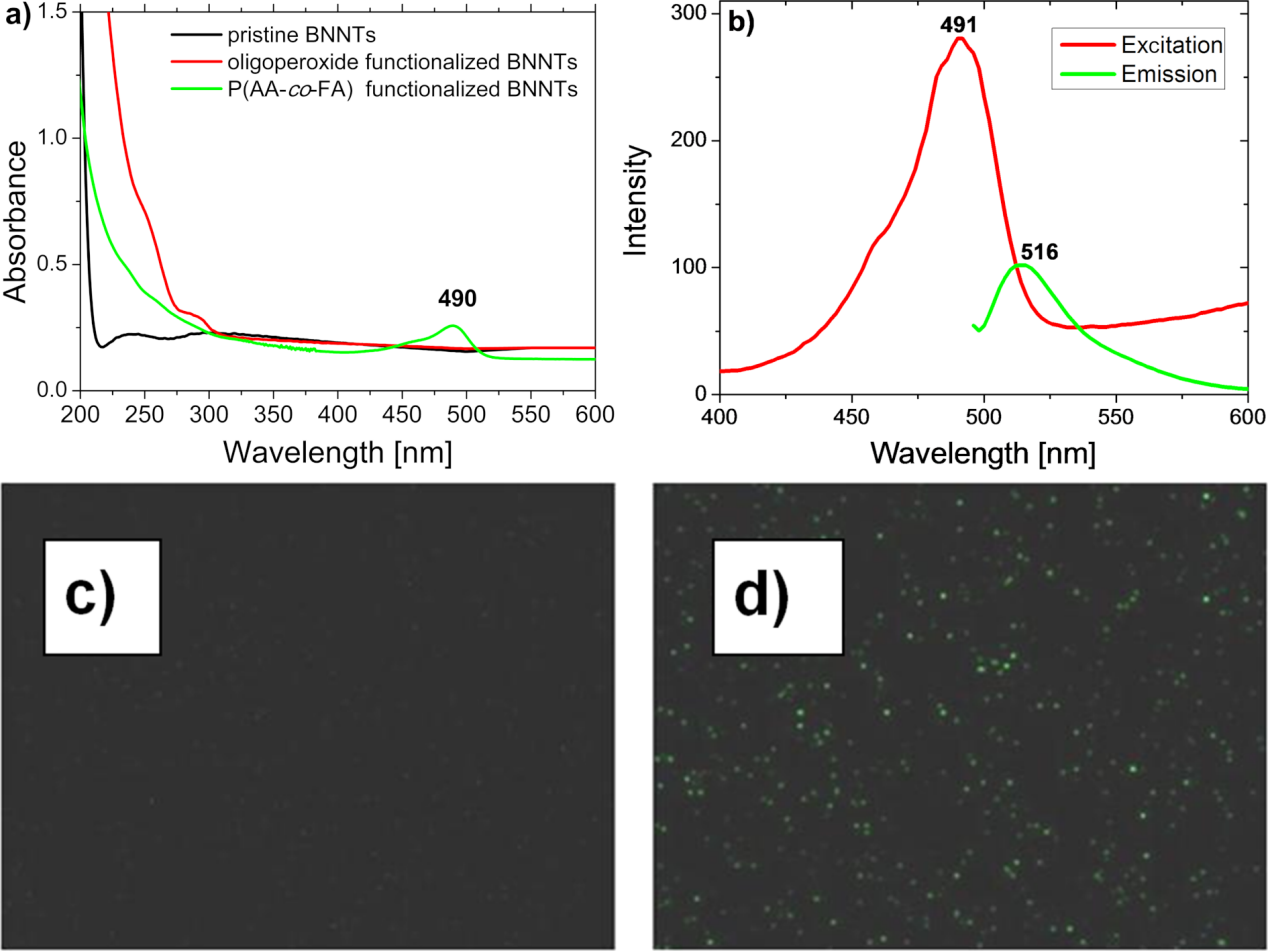
UV–vis spectra of BNNTs, oligoperoxide-functionalized BNNTs and P(AA-*co*-FA)-functionalized BNNTs (a) and fluorescence of P(AA-*co*-FA)-functionalized BNNTs (b). Laser scanning confocal microscope images of pure BNNT (c) and P(AA-*co*-FA)-functionalized BNNTs (d).

The optical properties of P(AA-*co*-FA)-functionalized BNNTs were studied by absorption and confocal microscopy. Figure 4a shows the absorption spectra of BNNTs, oligoperoxide-functionalized BNNTs, and P(AA-*co*-FA)-functionalized BNNTs dispersed in water. P(AA-*co*-FA)-functionalized BNNTs demonstrate a strong absorption band at λ = 490 nm, which is very close to the maximum adsorption peak of fluorescein. However, the pristine BNNTs and the oligoperoxide-functionalized BNNTs do not have absorption in this region.

Figure 4b shows the result of the fluorescence scanning of the P(AA-*co*-FA)-functionalized BNNTs. The fluorescence spectra of the P(AA-*co*-FA)-functionalized BNNTs retain the excitation maxima at 491 nm and the emission maximum at 516 nm, which is in good agreement with the literature [49]. In contrast to “pure” BNNTs, where fluorescence is absent (Figure 4c), P(AA-*co*-FA)-functionalized BNNTs (Figure 4d) demonstrate intense green fluorescence emission at 520 nm. The fluorescence spectra of the P(AA-*co*-FA)-functionalized BNNTs is important evidence for the grafting of P(AA-*co*-FA) on the BNNTs. These fluorescence properties are due to the π–π* transition in covalently bonded FA (chromophore) units of P(AA-*co*-FA)-functionalized BNNTs.

**Figure 5:**
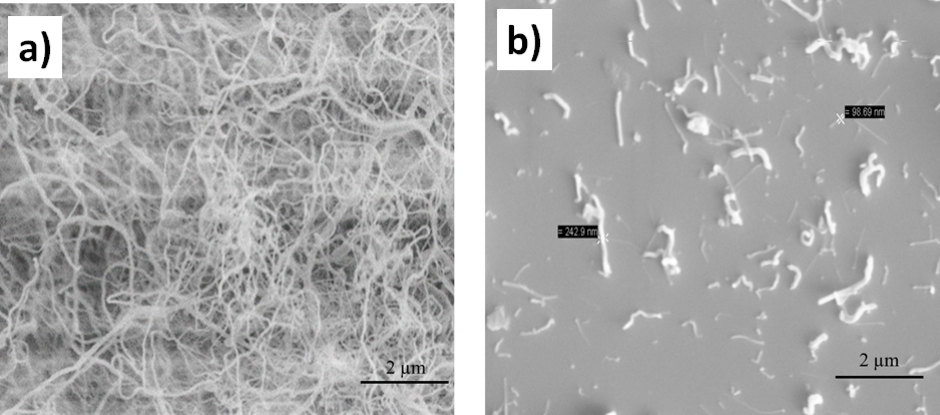
SEM image of pristine BNNTs (a) and P(AA-*co*-FA)-functionalized BNNTs (b) after dispersion in water.

**Figure 6:**
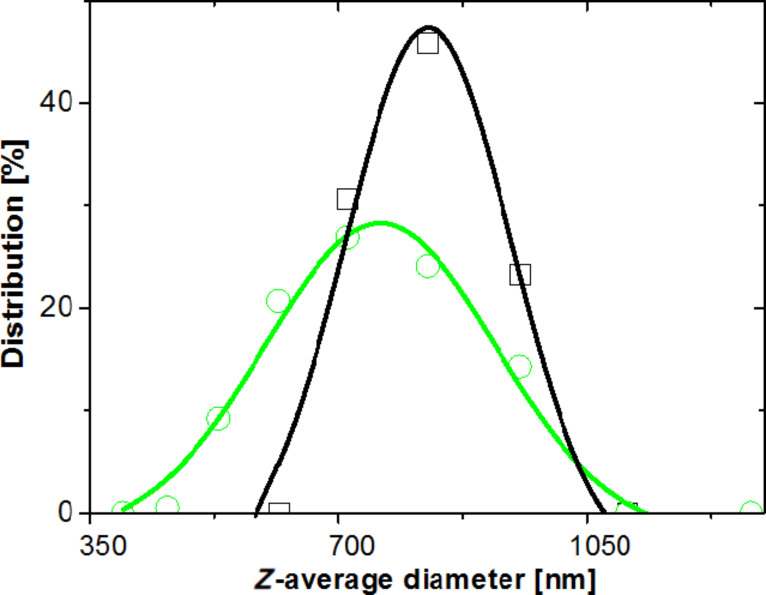
Size-distribution profile of BNNTs (black squares) and P(AA-*co*-FA)-functionalized BNNTs (green circles) in water. The concentration in the distilled water was 1 mg/mL.

SEM images of pure and P(AA-*co*-FA)-functionalized BNNTs after dispersion in water and drying on the surface are presented in Figure 5. The pristine BNNTs form large aggregates of several micrometers (Figure 5a). In contrast, the P(AA-*co*-FA)-functionalized BNNTs are highly dispersed in water and only single nanotubes are visible (Figure 5b). The size-distribution profile (Figure 6) and zeta potential of BNNTs and P(AA-*co*-FA)-functionalized BNNTs in water were also studied. P(AA-*co*-FA)-functionalized BNNTs demonstrate a smaller *Z*-average diameter than the native BNNTs. The zeta potentials of BNNTs and P(AA-*co*-FA)-functionalized BNNTs are −19.4 and −14.3 mV, respectively. The zeta potential of −17.7 mV found for the BNNTs modified by oligoperoxide is only slightly smaller than that of “native” BNNTs. The zeta potential value for BNNTs is completely in agreement with our previous result [50]. However, oligoperoxide and P(AA-*co*-FA) have numerous carboxylic groups in their structure and one can expect significant change upon functionalization. This drastic change can be explained by the physical coverage of the functional group in the polymeric structure during functionalization.

In light of these results, we can state that P(AA-*co*-FA)-functionalized BNNTs exhibit good dispersibility in water and their fluorescent properties make them excellent candidates for biomedical applications.

### pH-Responsive fluorescent properties of P(AA-*co*-FA)-functionalized BNNTs

**Scheme 2:**
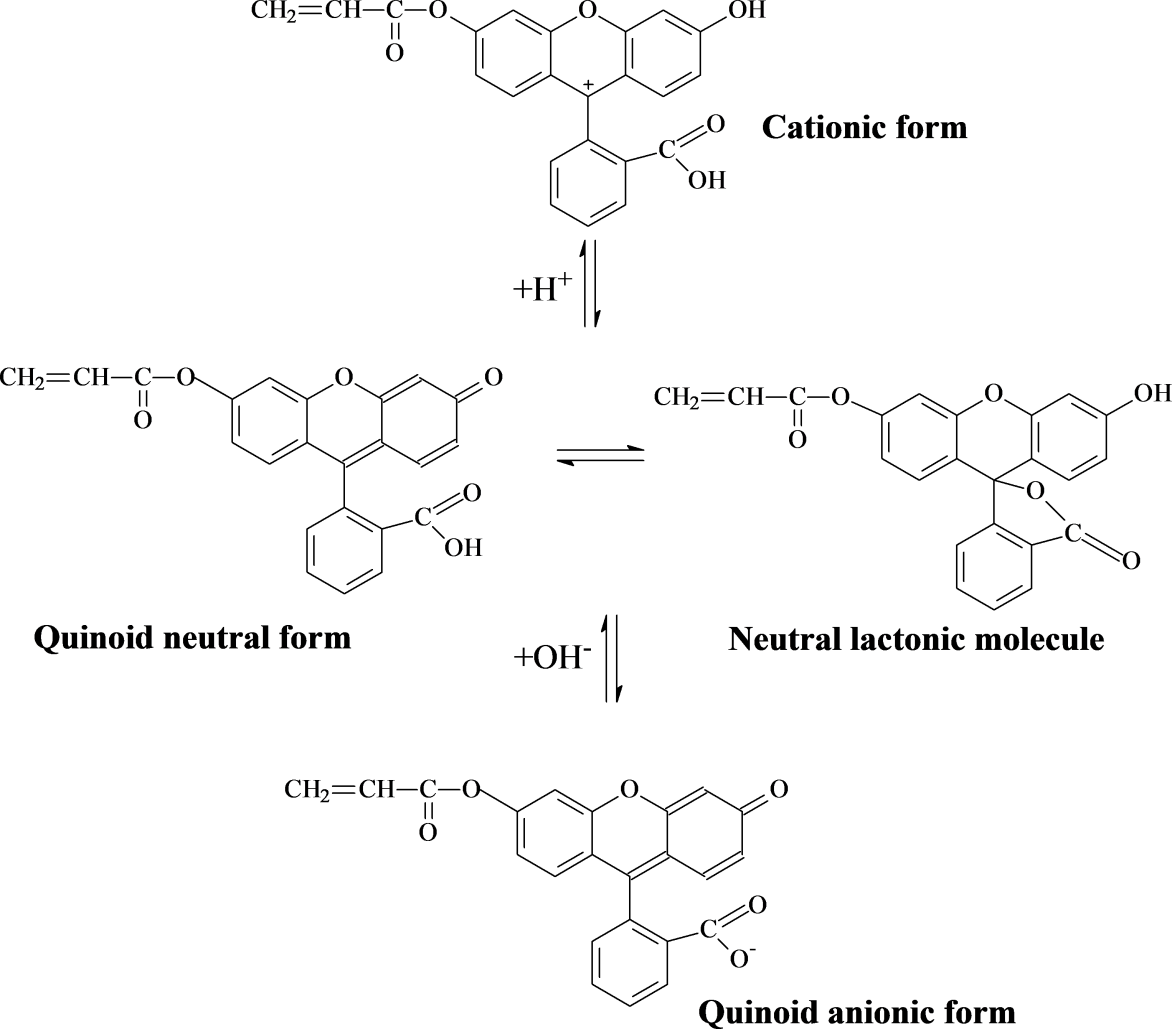
Forms of fluorescein acrylate at different pH [49].

Depending on pH, fluorescein can exist as a cation, monoanion, dianion, amphoion, neutral quinoid structure or neutral lactonic molecule [51]. Fluorescein monoacrylate, similar to fluorescein, can also form different molecular states (Scheme 2) depending upon pH [49,52]. Neutral and anionic forms of FMA units are responsible for fluorescence emission, but not the cationic form [51–52]. Fluorescein in a copolymer of hybrid P(AA-*co*-FA)-BNNT can be expected to show switching properties depending on pH.

**Figure 7:**
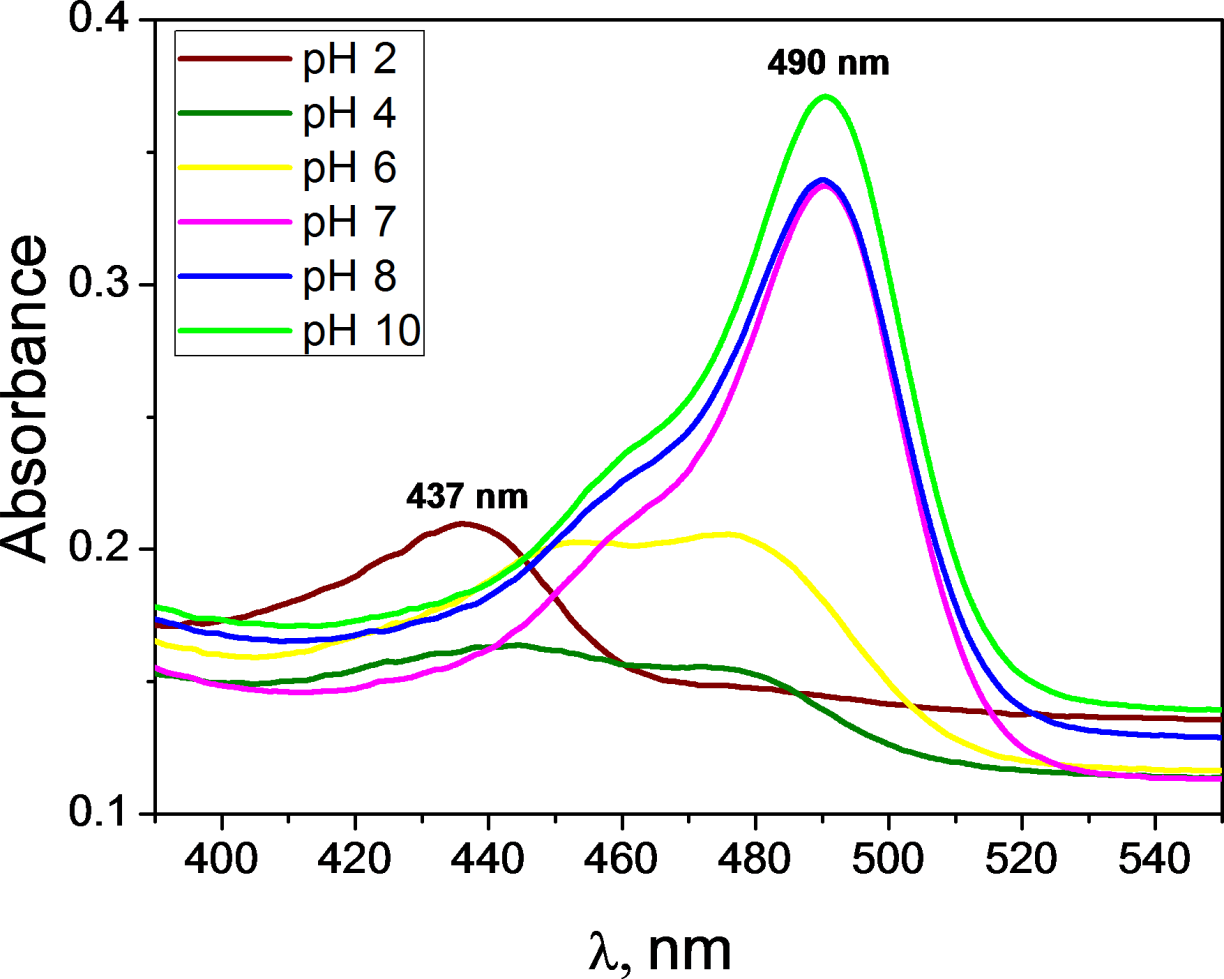
UV–vis spectra of a water suspension of P(AA-*co*-FA)-functionalized BNNTs (1 mg/mL) at various pH values.

Figure 7 shows the UV–vis spectra of a water-dispersed P(AA-*co*-FA)-functionalized BNNTs at various pH values. All samples have the same concentration. It is known from the literature [51] that in acidic media the maximum fluorescein absorption is at 437 nm but in alkaline solution it is at about 490 nm, and the absorption intensity is significantly higher than that of the acidic solution. As seen in the figure, the highest intensity of the absorbance is at 490 nm for pH 10 (Figure 7, green line) and slightly less at pH 7–8 (Figure 7, blue and red lines). In contrast, at pH 6 (Figure 7, yellow line) a strongly shifted, small, intense absorbance peak is observed. At pH 4 (Figure 7, green line) the absorbance is very poor, and we can state here that it has no absorbance. Only at pH 2 (Figure 7, brown line) do we observe a maximum fluorescein absorption at 437 nm [51].

**Figure 8:**
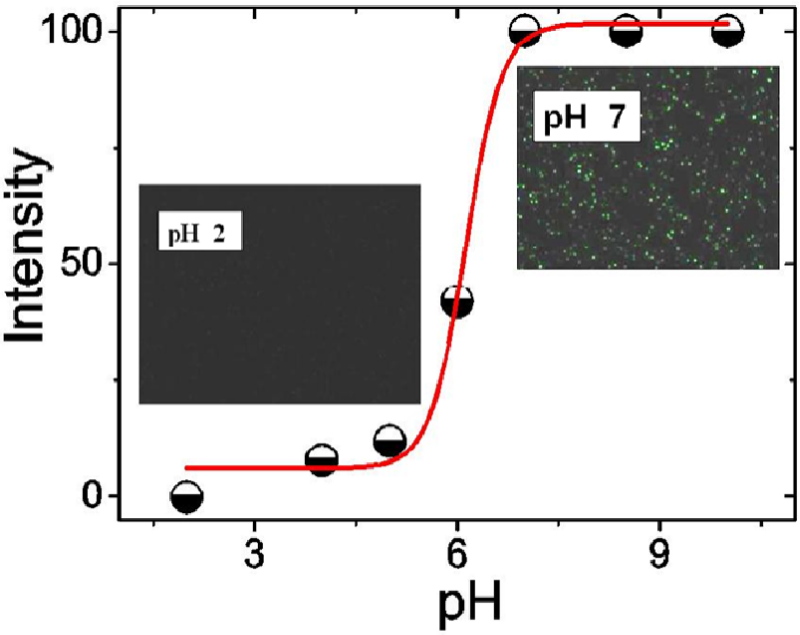
Fluorescence intensity of P(AA-*co*-FA)-functionalized BNNTs in solution under different pH conditions. The fluorescence intensity was measured in distilled water and the pH was adjusted by adding HCl or NaOH. The P(AA-*co*-FA)-functionalized BNNT concentration in the solution was (1 mg/mL). The images demonstrate the fluorescence intensity in the dry state at different pH values recorded by LSCM. Excitation wavelength – 490 nm, emission wavelength – 520 nm.

The typical fluorescence spectra of P(AA-*co*-FA)-functionalized BNNTs at various pH at different pH values are shown in Supporting Information File 1, Figure S1. The experiments were performed in the pH range of 2 to 10. Figure 8 shows the fluorescence intensity of P(AA-*co*-FA)-functionalized BNNTs. Under acidic conditions (pH 2–6), fluorescence molecules exist in neutral form and the emission spectra peaks of the P(AA-*co*-FA)-functionalized BNNTs are absent or of extremely low intensity (see Figure 8 and Figure S1 in Supporting Information File 1). Under neutral or alkaline pH values (pH 7–10), the P(AA-*co*-FA)-functionalized BNNTs are highly emissive, corresponding to the conjugated anionic form of the fluorescein monoacrylate molecule. The carboxylic group of fluorescein at neutral or alkaline pH values are deprotonated and become monoanionic fluorescein monoacrylate, whose electron density of the conjugated system for fluorescein molecules is very much enhanced, causing the fluorescence quantum yield to be much higher than that at acidic pH values [49]. No increase in the absorption intensity was observed for P(AA-*co*-FA)-functionalized BNNTs when the pH was elevated from 7 to 10, showing that the highest possible amount of the anionic form of fluorescein monoacrylate was reached at around pH 7.

We can assume that the influence of the pH-dependent changes of the conformation of the acrylic units in P(AA-*co*-FA)-grafted brushes on the fluorescent properties is rather weak in this system. The crucial role in pH-controlled switching of fluoresce belongs to the transformation of the fluorescein monoacrylate units from cationic form to neutral, and especially anionic forms. The conformation of the poly(acrylic acid) chain at acidic pH is not a collapsed structure and remains soluble in water [53]. However, pH-dependent changes of the conformation of the acrylic units in P(AA-*co*-FA)-grafted brushes can have a strong influence on the dispersibility of the functionalized-BNNTs in water. For acidic pH values, intermolecular aggregation of poly(acrylic acid) due to hydrophobic interactions was shown [54]. At neutral or basic pH, the hydrodynamic diameter of the poly(acrylic acid) macromolecules was signiﬁcantly decreased, indicating the disruption of intermolecular aggregates [54].

### Cellular imaging using P(AA-*co*-FA)-functionalized BNNTs

Fluorescence microscopy of living cells has become an integral part of modern cell biology. Most often cellular imaging is provided using fluorescent labels, including fluorescent dyes, nanoparticles, nanocomposites or proteins [55]. This label must meet certain criteria, such as biocompatibility, molecular recognition, chemical stability, and ability to be taken up in cells, etc. [55].

It is known that in living cells, both pH and temperature fluctuations are experienced during cellular processes such as endosytosis, gene expression, enzymatic reactions and metabolism [56–57]. Abnormal intracellular pH values are often associated with cell dysfunction and are observed in diseases such as cancer [58] and Alzheimer’s [59]. Therefore, information on intracellular pH is a crucial factor in understanding the physiological and pathological processes within living cells.

In our previous work, BNNTs were tested for their delivery potential, which showed promising results for imaging and drug targeting applications [60–61]. As a continuation of the work [60–61], we demonstrate the potential of BNNTs as a novel pH-switchable fluorescent water-dispersed material for cellular imaging.

**Figure 9:**
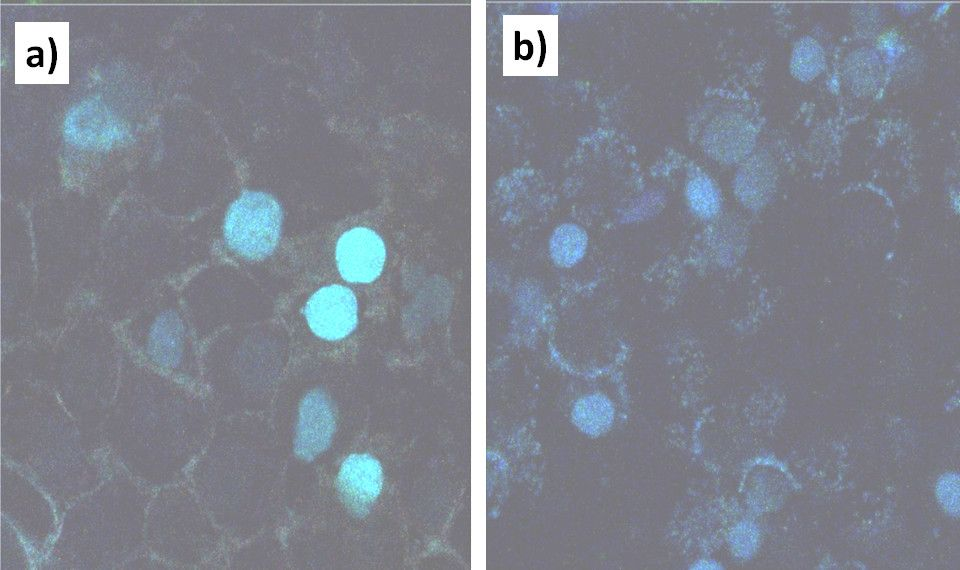
Fluorescent images recorded for human normal prostate epithelium cellular line (PNT1A) (a) and human prostate cancer cellular line (DU145) (b) with uptake of P(AA-*co*-FA)-functionalized BNNTs. Excitation wavelength: 490 nm; emission wavelength: 520 nm.

To study the uptake of P(AA-*co*-FA)-functionalized BNNTs into human normal prostate epithelium (PNT1A) and human prostate cancer (DU145) cell lines, we used fluorescence microscopy with excitation at 490 nm and emission at 520 nm (Figure 9). The autofluorescence of healthy cells and cancer cells was strictly avoided. DU145 cells internalize more P(AA-*co*-FA)-functionalized BNNTs because of their higher nutrition requirement for their fast proliferation and growth. While only P(AA-*co*-FA)-functionalized BNNT labeled cell nuclei were detectable in PNT1A control cells, the cells incubated with P(AA-*co*-FA)-functionalized BNNTs clearly showed fluorescence not only in the nuclei but also in the cytosol. The reason for this is likely due to the more internalized material compared to the healthy cells as a result of higher metabolic activity of cancer cells. This study clearly shows that the cellular uptake of P(AA-*co*-FA)-functionalized BNNTs is excellent and they can potentially be used in biomedical applications. We plan to continue to explore this new hybrid in our future studies not only as a pH-switchable label but also as “smart” surfaces and nanocarriers.

## Conclusion

pH-Switchable, fluorescent, hybrid, water-dispersed nanomaterials based on BNNTs and grafted brushes of copolymer P(AA-*co*-FA) were successfully fabricated in a two-step process and have several distinct advantages. First, the functionalized BNNTs in contrast to “native” BNNTs are highly dispersible in water. Second, the functionalized BNNTs demonstrate pH-dependent fluorescence properties. Third, acrylic groups in P(AA-*co*-FA) can be easy modified for drugs and/or targeting agents similar to that reported in [61]. It is well known that the carboxyl group of acrylic acid can react with ammonia to form acrylamide, or with an alcohol to form an acrylate ester. Moreover, the carboxyl group is able to create ionic bonds with numerous chemicals. In addition, it was shown that functionalized BNNTs are easily taken up by cells and could be considered as promising nanomaterials for cellular imaging and beyond.

The functionalized BNNTs were characterized using spectroscopic, gravimetric and imaging techniques, including FTIR, UV–vis, DLS, TGA, LSCM and SEM. The data clearly confirmed the functionalization of BNNTs with P(AA-*co*-FA). In contrast to “pure” BNNTs, P(AA-*co*-FA)-functionalized BNNTs demonstrate an intense green emission at 520 nm. Moreover, the pH-switchable fluorescence of the obtained BNNTs was previously described for fluorescein [46,49,51] and fluorescein-containing polymers [42,52]. Under neutral or alkaline pH values (pH 7–10), the P(AA-*co*-FA)-functionalized BNNTs are highly emissive, corresponding to the conjugated anionic form of fluorescein monoacrylate molecule. The carboxylic group of fluorescein at neutral or alkaline pH are deprotonated, forming monoanionic fluorescein monoacrylate, whose electron density of the conjugated system for fluorescein molecules is very much enhanced resulting in much higher quantum yield than that under acidic pH values [49]. No increase in the absorption intensity was observed for P(AA-*co*-FA)-functionalized BNNTs when the pH was elevated from 7 to 10, indicating that the highest possible concentration of the anionic form of fluorescein monoacrylate was reached at around pH 7.

Finally, P(AA-*co*-FA)-functionalized BNNTs were tested as a fluorescent label for cellular imaging. The functionalized BNNTs were easily taken up by PNT1A and DU145 cells and demonstrated good fluorescence properties. We concluded that P(AA-*co*-FA)-graft-oligoperoxide functionalized water-dispersed pH-switchable fluorescent BNNTs have great potential in biomedical applications as “smart” surfaces, nanocarriers and fluorescent labels.

## Supporting Information

File 1 Fluorescence scanning (typical excitation and emission spectra) of P(AA-*co*-FM)-functionalized BNNTs under different pH conditions.
