# Supplementary material for: pH-Controlled fluorescence switching in water-dispersed polymer brushes grafted to modified boron nitride nanotubes for cellular imaging
Source: Beilstein J Nanotechnol. 2019 Dec 10;10:2428–39. doi: 10.3762/bjnano.10.233 (PMC6941444; doi:10.3762/bjnano.10.233)
Supplement: File 1 — Fluorescence scanning (typical excitation and emission spectra) of P(AA-co-FM)-functionalized BNNTs under different pH conditions. [file Beilstein_J_Nanotechnol-10-2428-s001.pdf]

BEILSTEIN JOURNAL OF NANOTECHNOLOGY

Supporting Information

for

pH-Controlled fluorescence switching in water-dispersed polymer brushes grafted to modified boron nitride nanotubes for cellular imaging

Saban Kalay, Yurij Stetsyshyn, Volodymyr Donchak, Khrystyna Harhay, Ostap Lishchynskyi, Halyna Ohar, Yuriy Panchenko, Stanislav Voronov and Mustafa Çulha

Beilstein J. Nanotechnol. **2019,** *10,* 2428–2439. doi:10.3762/bjnano.10.233

Fluorescence scanning (typical excitation and emission spectra) of P(AA-*co*-FM)-functionalized BNNTs under different pH conditions

License and Terms: This is a supporting information file under the terms of the Creative Commons Attribution License (http://creativecommons.org/licenses/by/4.0). Please note that the reuse, redistribution and reproduction in particular requires that the authors and source are credited. The license is subject to the *Beilstein Journal of Nanotechnology* terms and conditions: (https://www.beilstein-journals.org/bjnano)

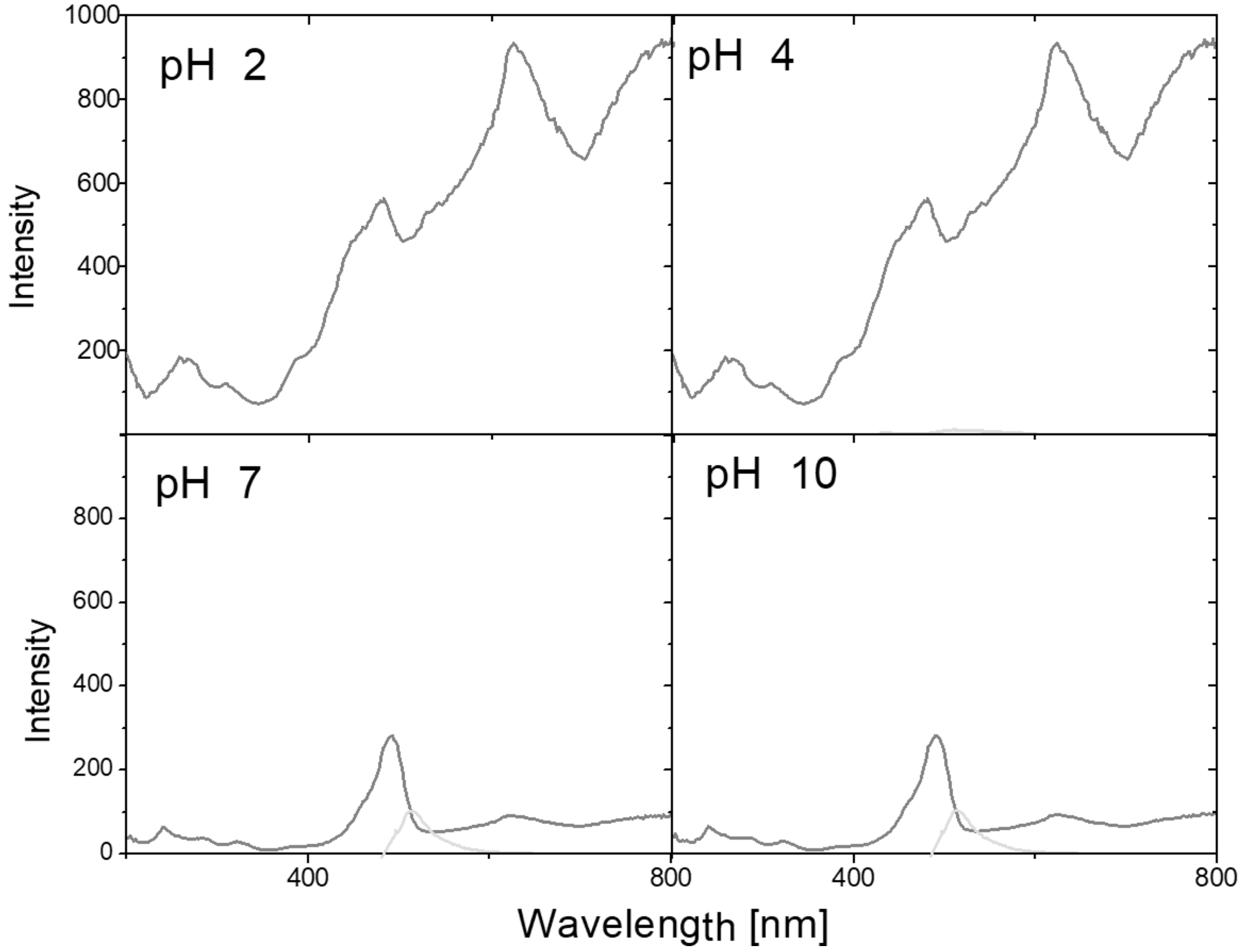

Figure S1: Fluorescence scanning (typical excitation and emission spectra) of P(AA-*co*-FM)-functionalized BNNTs for different pH values. The fluorescence intensity was measured in deionized water and the pH adjusted by adding HCl or NaOH. The P(AA-*co*-FA)-functionalized BNNT concentration in the solution was (1 mg/mL).
